# Profile of Selected Secondary Metabolites and Antioxidant Activity of Valerian and Lovage Grown in Organic and Low-Input Conventional System

**DOI:** 10.3390/metabo12090835

**Published:** 2022-09-03

**Authors:** Dominika Średnicka-Tober, Ewelina Hallmann, Klaudia Kopczyńska, Rita Góralska-Walczak, Marcin Barański, Alicja Grycz, Katarzyna Seidler-Łożykowska, Ewa Rembiałkowska, Renata Kazimierczak

**Affiliations:** 1 Department of Functional and Organic Food, Institute of Human Nutrition Sciences, Warsaw University of Life Sciences, Nowoursynowska 159c, 02-776 Warsaw, Poland; 2 Bioeconomy Research Institute, Agriculture Academy, Vytautas Magnus University, K. Donelaičio Str. 58, 44248 Kaunas, Lithuania; 3 Laboratory of Neurobiology, Nencki Institute of Experimental Biology, Polish Academy of Sciences, Pasteura 3, 02-093 Warsaw, Poland; 4 Medicinal Plants Breeding Research Laboratory, Institute of Natural Fibres and Medicinal Plants, Wojska Polskiego 71b, 60-630 Poznań, Poland

**Keywords:** medicinal plants, herbs, lovage, valerian, organic, conventional, low input, phenolic compounds, flavonoids, phenolic acids, antioxidant activity, HPLC

## Abstract

In the present study, the roots of valerian (*Valeriana officinalis* L.) and lovage (*Levisticum officinale* Koch.) from the organic and low-input conventional cultivation systems were subjected to the analysis of selected groups of phenolic compounds (phenolic acids, flavonoids) and antioxidant activity. Plants were grown in two consecutive vegetation seasons in the experimental plots located in western Poland. Phenolic acids and flavonoids were determined by high performance liquid chromatography (HPLC/UV–Vis), while the antioxidant activity of the samples was measured with the use of DPPH radical scavenging activity assay. The concentrations of phenolic acids (sum) and flavonoids (sum) were found to be higher in the conventional lovage roots, as compared to the organically grown lovage roots, while in the case of valerian, no significant effects of the cultivation system on the levels of the sums of these analyzed compounds were found. Furthermore, no significant effect of the cultivation system on the antioxidant activity of herbs was observed. Additional efforts could be invested in enhancing the potential of organic medicinal plants to consistently present the expected high concentrations of health-promoting antioxidants, which could be effectively brought through their post-harvest handling, storage and processing, and thus meet consumers’ expectations at the stage when they reach the market.

## 1. Introduction

Valerian (*Valeriana officinalis* L.) is a herb that belongs to the *Valerianaceae* family. This perennial plant grows natively in Europe and Asia and is also cultivated in North America. The Valerianaceae family consists of around 300 species. Valerian is mostly known for its sedative properties and for centuries, it has been used as a medicinal plant. There are various herbal remedies available on the market that contain dried root or extract from the root of valerian. It is widely used in the treatment of insomnia, anxiety, stress and nervous tension [1,2]; however, its usage is not limited to treating these disorders. Compounds found in *Valeriana officinalis* have a broad spectrum of biological activities, such as antioxidant, anti-inflammatory, antimicrobial, anxiolytic, antirheumatic, spasmolytic and neuroprotective activities, among others [3,4].

Although the health-promoting properties of valerian are considered to result from the joint action of a great number of its constituents [5], major focus of the research carried out to date has been given to the following two groups of phytochemicals: valepotriates (iridoid molecules) and sesquiterpenes—volatile compounds present in the essential oil of valerian, including mainly valerenal, valerenyl acetate, valerenic acid and valerenyl isovalerate [6,7]. Other previously studied bioactive components of valerian include choline, sterols and several alkaloids (actinidine, valerianine, valerine, and chatinine) [8].

Lovage (*Levisticum officinale* Koch.) is a herbaceous perennial plant that belongs to the *Apiaceae* family. It has been widely grown for centuries, due to its aromatic and ornamental values, but also medicinal properties. Lovage roots and leaves have been used for various medical purposes, due to their diuretic, apoptotic, antimycobacterial, estrogenic and spasmolytic activities [9,10,11]. Nowadays, lovage is utilized in cosmetic, food and beverages, perfumery and tobacco industries. It is also often used as a spice, previously shown to impact the regulation of the digestion and activation of enzymes that enhance nutrient absorption [11,12]. Lovage extractable oil, present in all parts of the plant, contains mainly phthalides (ligustilide, butylphthalide, sedanolide) and terpenoids, including n-butyl-phthalide, n-butylidene phthalide, sedanonic anhydride, D-terpineol, carvacrol, eugenol and volatile (essential) oil. The latter was found to be rich in, i.e., angelic acid and β-terpenol, but also coumarins, furocoumarins, including psoralins, rotoside, sitosterols and resins [13].

It should be pointed out that next to the compounds’ characteristic for these two species, both valerian and lovage contain numerous bioactive compounds that show antioxidant properties, such as phenolics and, among them, phenolic acids and flavonoids [8,14]. Many studies confirm that these groups of compounds have high antioxidant potential and anticarcinogenic, antibacterial, antiviral, anti-atherosclerotic and anti-inflammatory effects [15]. A high content of phenolic compounds in the diet has been considered as an important factor in the prevention of many non-communicable diseases related to free radical activity, such as cancers, cardiovascular disease, Parkinson’s disease, Alzheimer’s disease, atherosclerosis and many others [16,17,18].

In plants, phenolic compounds belong to the secondary metabolites involved in defense mechanisms. Their synthesis has been shown to be associated, among others, with pests’ pressure, exposure to UV light, bacterial and fungal infections and nutrients’ (i.e., nitrogen) availability during cultivation [18,19].

Nowadays, significant attention is given to organically produced plant-based foods. Their extensive production methods have been previously shown to result in several better quality and safety parameters compared to conventional, industrialized production [20]. Organic cultivation strategies rely on natural fertilizers and crop protection methods, as well as diverse crop rotations [21,22]. Even though several aspects of organic agriculture in relation to its environmental sustainability are still undergoing scientific discussion [23], environmental benefits of the organic systems have been broadly described in a number of research reports [24,25]. Many studies have also shown that crops grown in organic and other extensive agronomic systems are characterized by significantly higher concentrations of phenolic compounds and higher antioxidant activity than those grown with conventional practices [20,26,27], which is often linked to the limited availability of easily assimilable nitrogen and/or higher pressure of pests and diseases in organic cultivation [28,29].

Although organic cultivation of plants used for medicinal purposes should undoubtedly be an aspiration, the quality of medicinal plants from organic production is a relatively niche subject of research. A number of available studies that compare the concentrations of certain biologically active substances in medicinal plants from organic and conventional production is very limited, and, to the best of the authors’ knowledge, none of them focused on valerian (*Valeriana officinalis* L.) and/or lovage (*Levisticum officinale* Koch.) roots. The aim of this study was, therefore, to compare the content of two selected groups of phenolic compounds, phenolic acids and flavonoids, and the antioxidant activity of valerian and lovage grown in the organic and conventional cultivation system.

## 2. Materials and Methods

### 2.1. Research Material

The research material consisted of roots of valerian (*Valeriana officinalis* L.) cv. Polka and lovage (*Levisticum officinale* Koch.) cv. Amor cultivated in two consecutive seasons, according to the organic farming standards [21] and in the low-input conventional system, in the experimental plots of the Institute of Natural Fibres and Medicinal Plants located in Plewiska, western Poland (N 52°21′, E 16°48′). The experiment was established on the loamy soil of medium fertility, as a randomized-block design, on plots of 10 m^2^ area each, in three replications. All organic plots had been subjected to identical tillage and crop rotation schemes (oats with spring vetch–yellow lupine–rye with winter vetch) prior to the experiment. A moderate intensity of management, in line with good agricultural practice, was applied, meeting the high organic standards for medicinal plants. Manual weeding was used to control weeds. Neither pest nor disease protection was applied.

The conventional field was subjected to crop rotation schemes, including maize–maize–wheat. During the experiment, conventional plots were fertilized with mineral fertilizers, in doses corresponding to 40 kg N, 50 kg P (P_2_O_5)_ and 60 kg K (K_2_O). Phosphorus and potassium fertilizers were applied in autumn, while nitrogen fertilizer was applied in two doses—one at the beginning of the vegetation season (April) and the second in June/July. No chemical crop protection was used.

The seeds of valerian and lovage used to establish the experiment were obtained from the conservation breeding program carried out at the Institute of Natural Fibres and Medicinal Plants (Poznań, Poland). The seedlings of both plants were planted in May, with 45 cm spaces between rows, and 22.5 cm distance between plants in the row. The random samples were collected in autumn, by hand, from the area of approx. 1.0 m^2^ of each plot.

The collected raw materials were dried in natural conditions in the Institute of Natural Fibres and Medicinal Plants to reach 90.0 ± 2.0% of dry weight, and then transferred to the laboratory of the Department of Functional and Organic Food at the Warsaw University of Life Sciences (Warsaw, Poland). Before the analyses, the material was freeze-dried using a Labconco 2.5 freeze-dryer (Labconco Corporation, Kansas City, MO, USA) at a temperature of −40 ℃ and a pressure of 0.100 mBa, and ground in a laboratory mill A-11 (IKA^®^-Werke GmbH & Co. KG, Staufen im Breisgau, Germany). Ground samples were transferred into vials and kept at −80 ℃.

### 2.2. Phenolic Compound Extraction and Identification

Phenolic acids and flavonoids in the valerian and lovage samples were analyzed by high performance liquid chromatography (HPLC), using the Shimadzu HPLC system (USA Manufacturing Inc., Canby, OR, USA) with two LC-20AD pumps, a SIL-20AC autosampler, a CMB-20A system controller, a CTD-20AC oven, an ultraviolet–visible SPD-20AV detector and a Fusion-RP 80A column (250 mm × 4.60 mm, particle size: 4 µm) [30]. Freeze-dried powder was used to prepare the extract in 80% methanol. After 10 min in the ultrasonic bath (30 °C, 5500 Hz), the samples underwent centrifugation (5 min, 3180× *g*, 2 °C), and 1-mL aliquots of supernatant were transferred to HPLC vials. Water with acetonitrile (10% in Phase A and 55% in Phase B) were used as a gradient solvent in the HPLC analysis. The analysis lasted 36 min. The following gradient program was applied: 0–21.00 min: 95% Solvent A and 5% Solvent B; 21.01–25.00 min: 50% Solvent A and 50% Solvent B; 25.01–27.00 min: 20% Solvent A and 80% Solvent B; 27.01–32.00 min: 20% Solvent A and 80% Solvent B; and 32.01–36.00 min: 95% Solvent A and 5% Solvent B, with the flow rate of 1 mL min^−1^. The wavelengths of 270 and 360 nm were used for phenolic acid and flavonoid detection, respectively, and the column temperature was set at 30 °C. For individual phenolic acid and flavonoid identification, the Sigma-Aldrich and Fluka (Poznań, Poland) external standards with purities of 95.00–99.99% were used.

### 2.3. Antioxidant Activity

The antioxidant activity of the samples was measured with the use of DPPH radical scavenging activity assay [31]. It is based on the use of the stable free radical 2,2-diphenyl-1-picrylhydrazyl (DPPH), which is reduced in the presence of an antioxidant compound that acts as a hydrogen donor in the chemical reaction. The free radical DPPH is reduced to DPPH-H, which results in decolorization. The DPPH has a purple color and shows maximum absorbance at 517 nm. After reduction, it loses its color and becomes yellow and consequently, the absorbance decreases. Loss of color intensity is proportional to the amount of free radical unpaired electrons captured by the antioxidant. The larger the decrease in absorbance, the greater the antioxidant power.

Samples were weighted into the cuvettes, solvent was added, and the samples were placed in an ultrasonic bath for 15 min. Each sample was then shaken on vortex for 2 min and centrifuged for 8 min. Samples prepared as described above were pipetted onto the microplate, according to the ratio of 100 µL:200 µL between the extract and DPPH radical solution. The microplate was kept in a dark place for 15 min to incubate. After incubation of the samples, absorbance was measured in the spectrophotometer at 515 nm. For each measurement, a calibration curve for ascorbic acid standard was prepared, in order to verify if the samples’ absorbance results were in the range of the calibration points. To compare the different standards, the calibration curve for quercetin was also prepared. Both calibration curves were then used to calculate the final results, which were expressed as equivalents of the standards (ascorbic acid equivalents—AAE, quercetin equivalents—QE). The results were also presented as radical scavenging activity (RSA) [%], calculated on the basis of the following equation: RSA = (A_0_ − A_s_)/A_0_ × 100%, where A_0_ is the absorbance of DPPH radical solution and A_s_ is the absorbance of the solution containing the sample extract.

### 2.4. Statistical Analyses

All statistical analyses were performed in the R statistical environment [32]. In order to investigate the effect of the cultivation year and the production system on the measured composition parameters, two-way ANOVAs were derived from a linear mixed-effects model [33], which included the cultivation year and production system as the main factors, and the field location as a random factor. The normality of the residuals of all models was tested with the use of QQ-plots. The significance of the differences between the production system × cultivation year interaction means was additionally tested using Tukey contrasts in the general linear hypothesis testing (glht) function of the multcomp package in R [34]. A principal component analysis (PCA) was also performed to explore the possible differences and similarities in the composition of lovage and valerian samples grown in (a) different production systems and (b) in different cultivation years. The PCA was performed with the ‘prcomp’ function, and the plots were generated using the ‘ggbiplot’ package. Additionally, Pearson’s product-moment correlation analyses were carried out using the ‘cor’ function to identify potential linear associations between the concentrations of the individual and groups of the analyzed compounds in the lovage and valerian samples. The outcomes of the correlation analyses were visualized using the ‘corrplot’ package in R.

## 3. Results and Discussion

### 3.1. Phenolic Acid, Flavonoid and Antioxidant Activity of Lovage and Valerian Roots

**Figure 1:**
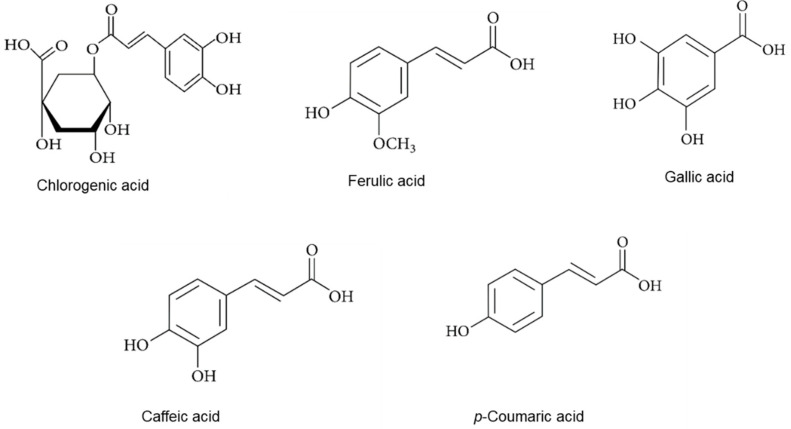
The chemical structures of phenolic acid identified in the lovage and valerian root samples.

**Figure 2:**
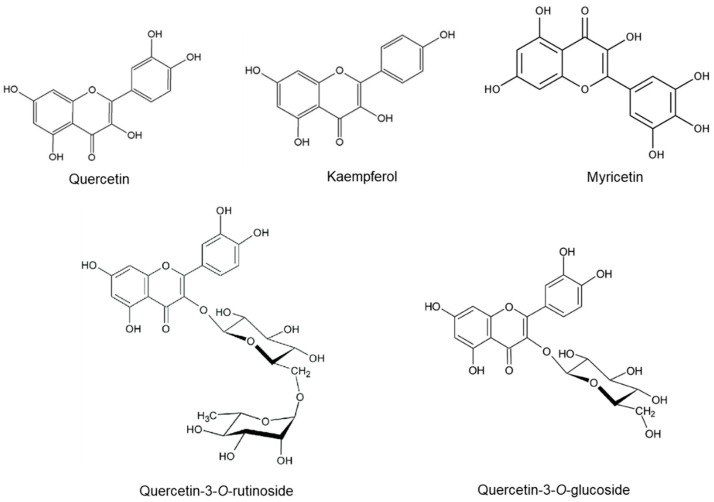
The chemical structures of flavonoids identified in the lovage and valerian root samples.

In the present study, concentrations of five phenolic acids and five flavonoid compounds were quantified in the tested lovage and valerian root samples. The chemical structures of the identified compounds are presented in Figure 1 (phenolic acids) and Figure 2 (flavonoids).

**Figure 3:**
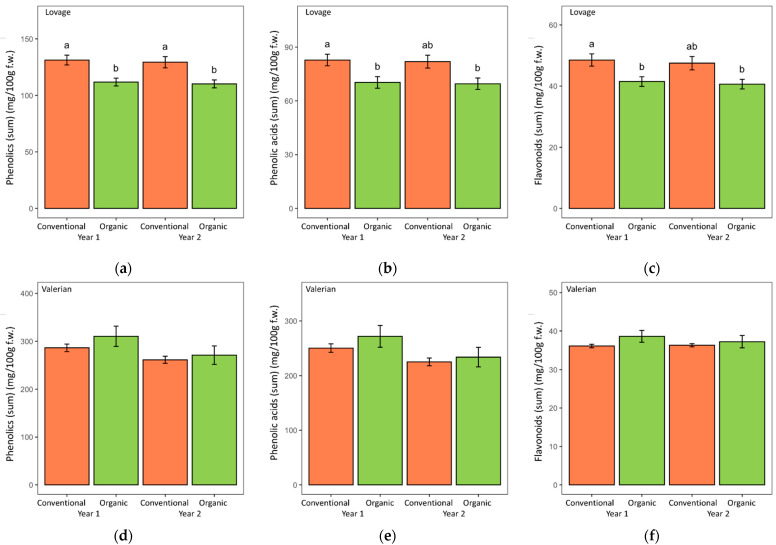
The content of phenolic compounds (sum), phenolic acids (sum) and flavonoids (sum) identified in lovage (**a**–**c**) and valerian (**d**–**f**) roots grown in two consecutive years in the organic and conventional system. Data are presented as means with standard errors. Within each figure plot, bars marked with different letters are significantly different at the 5% level of probability.

**Figure 4:**
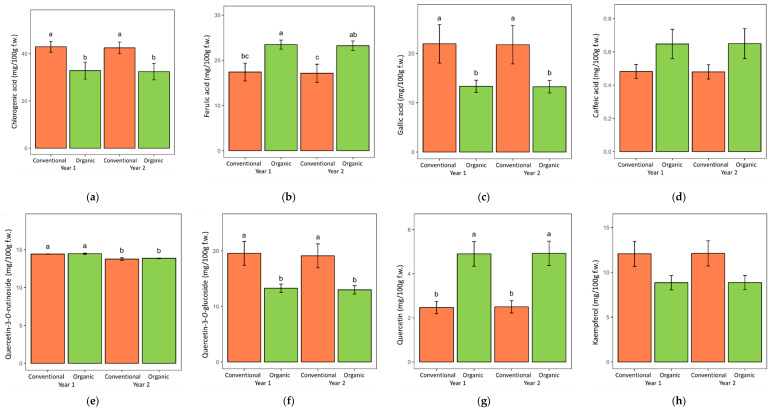
The content of each of the phenolic acids (**a**–**d**) and flavonoids (**e**–**h**) identified in lovage roots grown in two consecutive years in the organic and conventional system. Data are presented as means with standard errors. Within each figure plot, bars marked with different letters are significantly different at the 5% level of probability.

**Figure 5:**
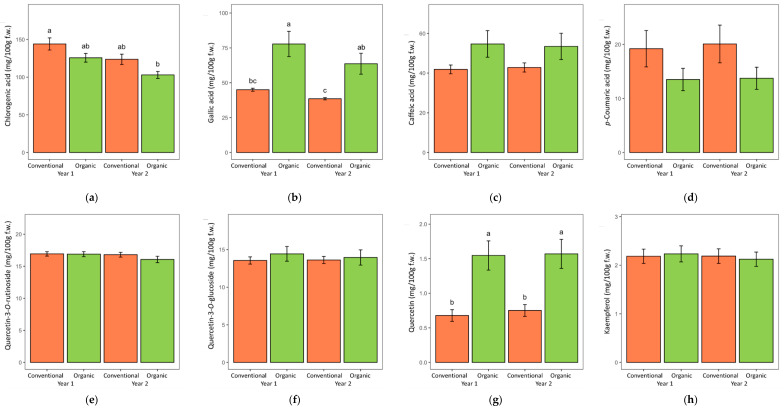
The content of each of the phenolic acids (**a**–**d**) and flavonoids (**e**–**h**) identified in valerian roots grown in two consecutive years in the organic and conventional system. Data are presented as means with standard errors. Within each figure plot, bars marked with different letters are significantly different at the 5% level of probability.

Table 1 and Table 2 and Figure 3, Figure 4 and Figure 5 show the concentrations of the tested phenolic acids and flavonoids in the lovage and valerian roots, depending on the cultivation system and year of the experiment. The two-factor ANOVA detected the significant effect of the cultivation system on phenolic (sum) and phenolic acid (sum) concentrations in lovage (*p* < 0.001), with significantly higher concentrations of these groups of compounds in the conventional roots, compared to the organically cultivated roots (Table 1). A high consistency of this trend in both years of the experiment was also observed (Figure 3). At the same time, valerian roots did not differ in the content of phenolics (sum), phenolic acids (sum) or flavonoids (sum) depending on the cultivation system (organic vs. conventional), but a significant effect of year was observed, with higher contents of phenolics (sum), and specifically phenolic acids (sum), in the first year compared to the second year of the experiment (Table 2).

When comparing the two herbs, valerian root appeared to be significantly richer in phenolic acids (225.14–274.13 mg/100 g f.w. vs. 81.1–82.78 mg/100 g f.w. in lovage), and consequently in phenolics (sum), while lovage roots contained significantly more flavonoids (43.16–48.53 mg/100 g f.w. vs. 36.09–37.26 mg/100 g f.w. in valerian).

When looking into the individual phenolic acid concentrations in the roots of both herbs, the following significant effects of the cultivation system were detected: lovage roots grown in the organic system contained more ferulic and caffeic acid and less chlorogenic and gallic acid compared to the conventionally grown lovage (Table 1, Figure 4), while valerian roots grown in the organic system contained more gallic and caffeic acid and less chlorogenic and *p*-coumaric acid than those grown in the conventional system (Table 2, Figure 5). When comparing samples from the two years of the experiment, lovage root samples from both years contained similar levels of individual phenolic acids (Table 1), while valerian roots from the first year were significantly richer in chlorogenic and gallic acid than in the following year (Table 2).

Quercetin-3-*O*-rutinoside and quercetin-3-*O*-glucoside were the main detected flavonoid compounds in the tested herbs (Table 1 and Table 2). Other flavonoids found in lower concentrations in the tested samples included quercetin and kaempferol (in both lovage and valerian) and myricetin (in valerian). The ANOVA detected a significant effect of the cultivation system on the concentration of quercetin-3-*O*-glucoside and kaempferol in lovage (significantly higher in conventionally grown roots) and quercetin (significantly higher in organically grown roots of both herbs). These differences were consistent in both years of the experiment (Figure 4 and Figure 5).

The outcome that there were no consistent effects of the cultivation system (organic vs. conventional) on the concentrations of the analyzed phenolic compounds in the tested samples of herbs was unexpected, since extensive meta-analyses that compare the compositions of organic and conventional vegetables and fruits have reported overall higher levels of many groups of antioxidants, including phenolic acids and flavonoids, in organic crops [20,35]. However, it is important to point out that the mentioned meta-analyses also detected a considerable variation among individual studies and crop types/species, and none of the original studies included in these meta-analyses specifically targeted lovage and/or valerian as study objects.

The availability of nitrogen as well as the irradiation intensity were previously shown as significant agronomic and environmental factors that impacted phenolics synthesis and concentration in crops, with higher phenolic contents being linked to limited nitrogen availability, typical for the organic systems [28,36]. However, in the study reported here, the nitrogen availability pattern was not controlled; thus, we could not investigate the relations between this potential explanatory factor and the level of phenolic compounds. Pressure of diseases and crop pests are also considered as potential factors that can trigger phenolic synthesis and result in higher concentrations of these compounds in plants, since phenolics are known to play an important role in the plant resistance response to biotrophic stresses [37]. The effect of year and cultivation system on the concentrations of some of the measured compounds could, therefore, have been due to differences in pest and disease pressure in both years and under both cultivation regimens. Higher disease and pest incidence was previously indicated as the potential reason for higher concentrations of phenolic and other resistance-related chemicals in organic compared to conventional crops [29], even though this has not been confirmed in the controlled experiments [28,36]. It is important to point out that in the present field experiment, no chemical crop protection was used in any of the two compared production systems. Thus, potential differences in the pests and diseases pressure between the two systems could result from, i.e., the differences in the fertility management applied and the crop rotation schemes. It was previously reported that diversifying crop rotation, typical for the organic agronomic systems, improves system robustness through enhancing crop resistance to and resilience from biotic-induced disturbances [38].

In the present study, only roots of the two medicinal plants underwent the analyses of phenolic acid and flavonoid content. Another study of lovage plants indicated that the phenolic acid concentrations in various plant parts were as follows: roots 0.12–0.16%, herb 0.88–1.03%, stems 0.30–0.39%, leaf 1.11–1.23% and fruits 1.32–1.41% [14].

**Figure 6:**
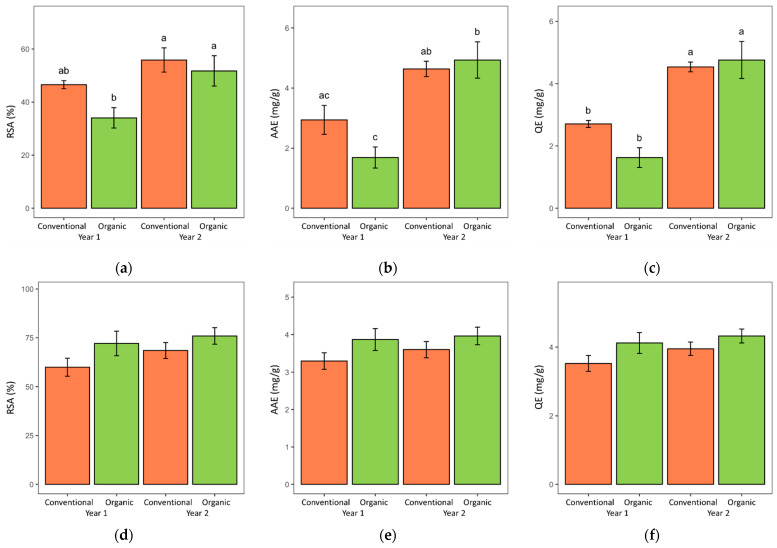
The antioxidant activity of dried lovage (**a**–**c**) and valerian (**d**–**f**) roots grown in two consecutive years in the organic and conventional system; expressed as: RSA—radical scavenging activity; AAE—ascorbic acid equivalents; quercetin equivalents—QE. Data are presented as means with standard errors. Within each figure plot, bars marked with different letters are significantly different at the 5% level of probability.

Table 3 and Figure 6 show the results on the antioxidant activity of the lovage and valerian root samples expressed as the radical scavenging activity (RSA), equivalents of ascorbic acid (AAE) and equivalents of quercetin (QE). Independent from the expression of the results, no significant cultivation system-related differences in the antioxidant activity of samples were detected (Table 3). Interestingly, while valerian samples from both years were characterized as rather stable values of the measured parameters, the lovage samples showed considerable between-year variation in all the three antioxidant activity measures (expressions), with considerably higher values in the second year compared to the first year. Moreover, the year-to-year differences were more pronounced in the case of organic lovage samples than the conventional lovage samples (Figure 6).

The authors of the previously mentioned meta-analyses [20] have identified a large number (over 150) of comparison studies that look into the antioxidant activity of various organic and conventional crops, with a range of different methodologies. Both weighted and unweighted meta-analyses confirmed a significantly higher antioxidant activity, on average, in organically grown crops. However, a separate analysis of data obtained for fruits and vegetables detected a significant difference for fruits, but only a trend towards such a difference in the case of vegetables. Moreover, the power of the detected differences varied largely depending on the specific antioxidant activity assays used. Again, none of the studies included in the mentioned meta-analysis focused on the antioxidant activity of organic vs. conventional medicinal plants, such as lovage and valerian roots.

Overall, the concentrations of antioxidants (i.e., phenolics) and the antioxidant activity of plants are known to be variable, depending, among others, on plant genotype, cultivation conditions and agronomic practices applied during cultivation, but also further post-harvest handling [39,40,41,42]. The results presented in this study, as well as those reported by other researchers, show that there is a need for investigating the best conditions and practices, increasing the crops’ potential for reaching high levels of health-promoting bioactive compounds.

### 3.2. Associations between Composition Data of the Tested Medicinal Plants

**Figure 7:**
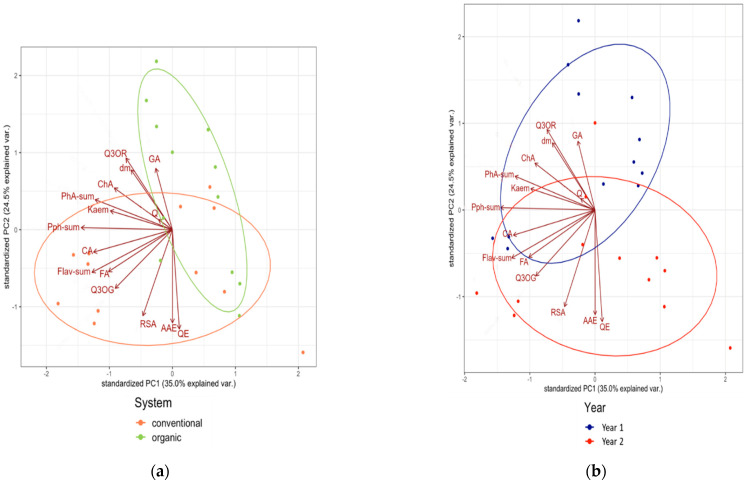
Principal component analysis (PCA) biplot, showing the relationships between the chemical composition of the dried lovage roots and (**a**) cultivation system and (**b**) year. AAE—ascorbic acid equivalents, CA—caffeic acid, ChA—chlorogenic acid, dm—dry matter, FA—ferulic acid, Flav-sum—flavonoids (sum), GA—gallic acid, Kaem—kaempferol, PhA-sum—phenolic acids (sum), Pph-sum—polyphenols (sum), Q—quercetin, Q3OG—quercetin-3-*O*-glucoside, Q3OR—quercetin-3-*O*-rutinoside, QE—quercetin equivalents, RSA—radical scavenging activity; PC1—the first principal component, PC2—the second principal component.

**Figure 8:**
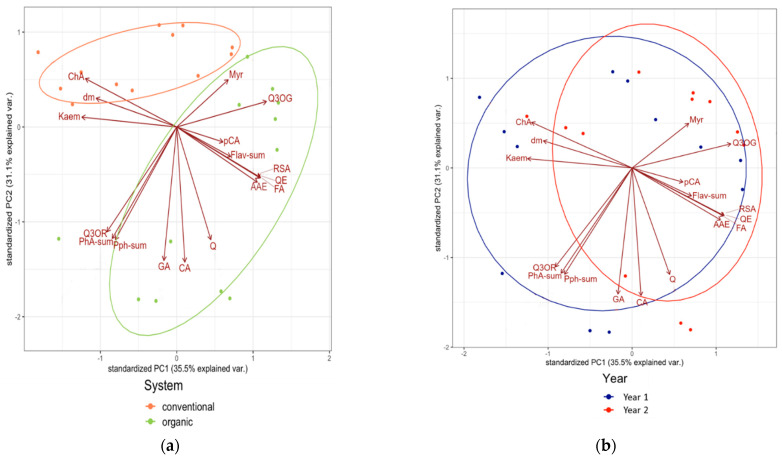
Principal component analysis (PCA) biplot showing the relationships between the chemical composition of the dried valerian roots and (**a**) cultivation system and (**b**) year. AAE—ascorbic acid equivalents, CA—caffeic acid, ChA—chlorogenic acid, dm—dry matter, Flav-sum—flavonoids (sum), GA—gallic acid, Kaem—kaempferol, Myr—myricetin, pCA—*p*-coumaric acid, PhA-sum—phenolic acids (sum), Pph-sum—polyphenols (sum), Q—quercetin, Q3OG—quercetin-3-*O*-glucoside, Q3OR—quercetin-3-*O*-rutinoside, QE—quercetin equivalents, RSA—radical scavenging activity; PC1—the first principal component and PC2—the second principal component.

Principal component analysis (PCA) was performed to further explore the possible differences and similarities in the composition of lovage and valerian roots grown in different cultivation systems (Figure 7a and Figure 8a) and in two years (Figure 7b and Figure 8b). The analysis demonstrated that the cultivation system resulted in the clearer separation of the results and explained a larger proportion of the variation than climatic differences between the two growing years. This was especially evident in the case of valerian (Figure 8). The PCA plot also shows a positive association between the organic cultivation system and concentrations of the majority of the tested parameters (except for chlorogenic acid and kaempferol concentration) in valerian (Figure 8a). A similar association was not observed in the case of lovage (Figure 7a).

**Figure 9:**
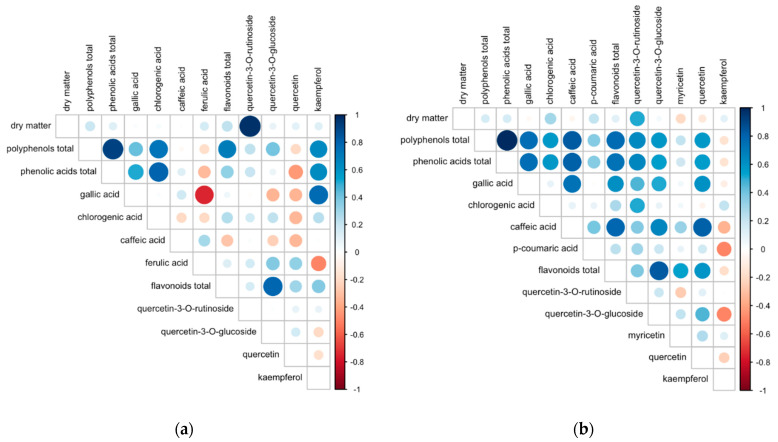
Pearson’s correlations between the concentrations of the analyzed compounds identified in (**a**) dried lovage roots and (**b**) dried valerian roots. Color (red/blue) and the color intensity indicate the direction and the strength of the association, while size of the circle reflects statistical significance of the correlation (*p*-value).

Pearson’s analysis detected a range of significant correlations between the composition parameters analyzed in both species of herbs (Figure 9a,b). In lovage, there was a strong negative correlation between gallic acid and ferulic acid content (r = −0.74, *p* < 0.001) and a strong positive correlation between gallic acid and kaempferol (r = 0.77, *p* < 0.001). In valerian, positive associations were found between the content of gallic acid and caffeic acid (r = 0.74, *p* < 0.001), quercetin-3-*O*-rutinoside (r = 0.46, *p* < 0.001), quercetin-3-*O*-glucoside (r = 0.52, *p* < 0.001) and quercetin (r = 0.60, *p* < 0.001). Similar positive associations were detected between the contents of caffeic acid and flavonoids, such as quercetin (r = 0.82, *p* < 0.001) and quercetin-3-*O*-glucoside (r = 0.67, *p* < 0.001). At the same time, negative associations were found between the concentrations of kaempferol and compounds, such as *p*-coumaric acid (r = −0.49, *p* < 0.001) and quercetin-3-*O*-glucoside (r = −0.50, *p* < 0.001). The detected interrelations could be explained by the biosynthesis and/or metabolism of the listed flavonoid compounds and phenolic acids being closely linked, but also by the fact that they are regulated by the same agronomic and environmental factors [43,44].

## 4. Conclusions

The present study shows that organic cultivation practices do not guarantee higher concentrations of phenolic acids and flavonoids or higher antioxidant capacity of lovage and valerian, when compared to the low-input (no chemical protection) conventional cultivation system. Additional efforts are needed to enhance the potential of organic medicinal plants to consistently present the expected high concentrations of health-promoting antioxidants, which could be effectively brought through their post-harvest handling, storage and processing, and thus meet consumers’ expectations at the stage when they reach the market. Performing research that could allow us to identify and promote strategies and specific factors to enhance the health-promoting qualities of medicinal plants grown in alternative, more sustainable systems is of interest for producers and consumers, who are increasingly searching for produce with high quality features and a low environmental footprint.

## Figures and Tables

**Table 1:** The effects of the production system and cultivation year on phenolic (sum), phenolic acid and flavonoid concentrations in dried roots of lovage.

	Phenolics (Sum)	Phenolic Acids (Sum)	Chlorogenic Acid	Ferulic Acid	Gallic Acid	Caffeic Acid	Flavonoids (Sum)	Quercetin-3-*O*- Rutinoside	Quercetin-3-*O*- Glucoside	Quercetin	Kaempferol
	mg/100 g f.w.										
System (SYS)											
Conventional	130 ± 3 ^1^	82.3 ± 2.4	42.7 ± 1.6	17.3 ± 1.4	21.9 ± 2.7	0.481 ± 0.03	48.0 ± 1.5	14.1 ± 0.1	19.3 ± 1.5	2.48 ± 0.19	12.1 ± 1.0
Organic	111 ± 2	69.9 ± 2.2	32.6 ± 2.5	23.4 ± 0.7	13.3 ± 0.9	0.648 ± 0.06	41.1 ± 1.1	14.2 ± 0.1	13.1 ± 0.5	4.91 ± 0.39	8.9 ± 0.6
Year (YR)											
Year 1	122 ± 3	76.5 ± 2.5	37.9 ± 2.3	20.4 ± 1.2	17.6 ± 2.1	0.565 ± 0.05	45.0 ± 1.4	14.4 ± 0.0	16.4 ± 1.2	3.68 ± 0.37	10.5 ± 0.8
Year 2	120 ± 3	75.7 ± 2.6	37.4 ± 2.3	20.2 ± 1.2	17.5 ± 2.1	0.564 ± 0.05	44.1 ± 1.5	13.8 ± 0.1	16.1 ± 1.2	3.71 ± 0.37	10.5 ± 0.8
ANOVA *p*-values											
SYS	**0.000** ^2^	**0.000**	**0.000**	**0.000**	**0.003**	**0.016**	**0.000**	0.482	**0.000**	**0.000**	**0.006**
YR	0.641	0.788	0.866	0.858	0.963	0.998	0.610	**0.000**	0.818	0.947	0.979
SYS × YR	0.965	0.979	0.996	0.995	0.984	0.976	0.963	0.781	0.960	0.990	0.989

^1^ Data are presented as means ± standard errors; ^2^ significant ANOVA *p*-values (*p* < 0.05) are marked in bold.

**Table 2:** The effects of the production system and cultivation year on phenolics (sum), phenolic acids and flavonoids concentrations in dried roots of valerian.

	Phenolics (Sum)	Phenolic Acids (Sum)	Chlorogenic Acid	Gallic Acid	Caffeic Acid	*p*-Coumaric Acid	Flavonoids (Sum)	Quercetin-3-*O*-Rutinoside	Quercetin-3-*O*-Glucoside	Quercetin	Kaempfe-Rol	Myricetin
	mg/100 g f.w.											
System (SYS)												
Conventional	274 ± 6 ^1^	238 ± 6	134 ± 5	41.8 ± 0.9	42.3 ± 1.6	19.7 ± 2.4	36.2 ± 0.3	16.9 ± 0.3	13.6 ± 0.3	0.71 ± 0.06	2.19 ± 0.10	2.84 ± 0.21
Organic	291 ± 14	253 ± 14	114 ± 4	70.7 ± 5.9	54.1 ± 4.6	13.6 ± 1.4	37.9 ± 1.1	16.5 ± 0.3	14.2 ± 0.7	1.56 ± 0.15	2.18 ± 0.11	3.51 ± 0.45
Year (YR)												
Year 1	298 ± 11	261 ± 11	135 ± 5	61.4 ± 5.3	48.3 ± 3.6	16.4 ± 2.0	37.4 ± 0.8	16.9 ± 0.3	14.0 ± 0.5	1.11 ± 0.13	2.21 ± 0.11	3.13 ± 0.37
Year 2	266 ± 10	229 ± 9	113 ± 4	51.1 ± 4.3	48.1 ± 3.6	16.9 ± 2.1	36.8 ± 0.8	16.4 ± 0.3	13.8 ± 0.6	1.16 ± 0.13	2.16 ± 0.10	3.22 ± 0.35
ANOVA *p*-values												
SYS	0.238	0.263	**0.004**	**0.000**	**0.011**	**0.029**	0.107	0.337	0.385	**0.000**	0.962	0.188
YR	**0.027 ^2^**	**0.022**	**0.001**	**0.030**	0.977	0.837	0.571	0.238	0.771	0.746	0.728	0.849
SYS × YR	0.609	0.631	0.857	0.408	0.806	0.905	0.443	0.391	0.697	0.869	0.711	0.834

^1^ Data are presented as means ± standard errors; ^2^ significant ANOVA *p*-values (*p* < 0.05) are marked in bold.

**Table 3:** The main effects of, and interactions between, the production system (conventional and organic) and cultivation year on the antioxidant activity of dried lovage and valerian roots.

	Lovage			Valerian		
	RSA ^1^ (%)	AAE ^2^ (mg/g)	QE ^3^ (mg/g)	RSA (%)	AAE (mg/g)	QE (mg/g)
System (SYS)						
Conventional	51.2 ± 2.7 ^4^	3.79 ± 0.36	3.62 ± 0.29	64.2 ± 3.2	3.45 ± 0.15	3.74 ± 0.16
Organic	42.9 ± 4.2	3.31 ± 0.59	3.19 ± 0.57	74.0 ± 3.6	3.91 ± 0.18	4.22 ± 0.18
Year (YR)						
Year 1	40.3 ± 2.7	2.31 ± 0.34	2.17 ± 0.23	66.1 ± 4.1	3.58 ± 0.19	3.82 ± 0.20
Year 2	53.8 ± 3.5	4.79 ± 0.32	4.65 ± 0.29	72.2 ± 3.0	3.78 ± 0.16	4.14 ± 0.15
ANOVA *p*-values						
SYS	0.053	0.295	0.234	0.059	0.069	0.055
YR	**0.003** ^5^	**0.000**	**0.000**	0.222	0.421	0.199
SYS × YR	0.308	0.097	0.078	0.640	0.674	0.649

^1^ RSA—radical scavenging activity; ^2^ AAE—ascorbic acid equivalents; ^3^ quercetin equivalents—QE; ^4^ data are presented as means ± standard errors; ^5^ significant ANOVA *p*-values (*p* < 0.05) are marked in bold.

## Data Availability

Data will be made available upon reasonable request by authors Dominika Średnicka-Tober and Renata Kazimierczak.
